# Nanoligomers targeting NF-κB and NLRP3 reduce neuroinflammation and improve cognitive function with aging and tauopathy

**DOI:** 10.1186/s12974-024-03182-9

**Published:** 2024-07-27

**Authors:** Devin Wahl, Sydney J. Risen, Shelby C. Osburn, Tobias Emge, Sadhana Sharma, Vincenzo S. Gilberto, Anushree Chatterjee, Prashant Nagpal, Julie A. Moreno, Thomas J. LaRocca

**Affiliations:** 1 Department of Health and Exercise Science, Colorado State University, 1582 Campus Delivery, Fort Collins, CO 80523 USA; 2 Columbine Health Systems Center for Healthy Aging, Colorado State University, Fort Collins, CO USA; 3 Department of Environmental and Radiological Health Sciences, Colorado State University, Fort Collins, CO USA; 4 Brain Research Center, Colorado State University, Fort Collins, CO USA; 5 Sachi Bio, Colorado Technology Center, Louisville, CO USA

**Keywords:** NF-κB, NLRP3, Neuroinflammation, Aging, Tauopathy, Cognitive function

## Abstract

Neuroinflammation contributes to impaired cognitive function in brain aging and neurodegenerative disorders like Alzheimer’s disease, which is characterized by the aggregation of pathological tau. One major driver of both age- and tau-associated neuroinflammation is the NF-κB and NLRP3 signaling axis. However, current treatments targeting NF-κB or NLRP3 may have adverse/systemic effects, and most have not been clinically translatable. In this study, we tested the efficacy of a novel, nucleic acid therapeutic (Nanoligomer) cocktail specifically targeting both NF-κB and NLRP3 in the brain for reducing neuroinflammation and improving cognitive function in old (aged 19 months) wildtype mice, and in rTg4510 tau pathology mice (aged 2 months). We found that 4 weeks of NF-κB/NLRP3-targeting Nanoligomer treatment strongly reduced neuro-inflammatory cytokine profiles in the brain and improved cognitive-behavioral function in both old and rTg4510 mice. These effects of NF-κB/NLRP3-targeting Nanoligomers were also associated with reduced glial cell activation and pathology, favorable changes in transcriptome signatures of glia-associated inflammation (reduced) and neuronal health (increased), and positive systemic effects. Collectively, our results provide a basis for future translational studies targeting both NF-κB and NLRP3 in the brain, perhaps using Nanoligomers, to inhibit neuroinflammation and improve cognitive function with aging and neurodegeneration.

**Supplementary Information:**

The online version contains supplementary material available at 10.1186/s12974-024-03182-9.

## Introduction

Aging is associated with declines in cognitive function, and it is the primary risk factor for most neurodegenerative diseases, including Alzheimer’s disease (AD). These declines in cognitive function are closely linked with the accumulation of pathological tau, a hallmark of AD that also increases with aging [1, 2]. One key “macro-mechanism” that contributes to both brain aging and tau pathology is neuroinflammation, which is characterized by innate immune signaling, glial cell activation, reduced neuronal health and the release of neurotoxic, pro-inflammatory cytokines [3]. In fact, evidence suggests that neuroinflammation may occur early in brain aging and both precede and be exacerbated by tauopathy [4]. As such, identifying strategies to reduce neuroinflammation in the context of aging and tauopathy is an important goal [5].

**Fig. 1:**
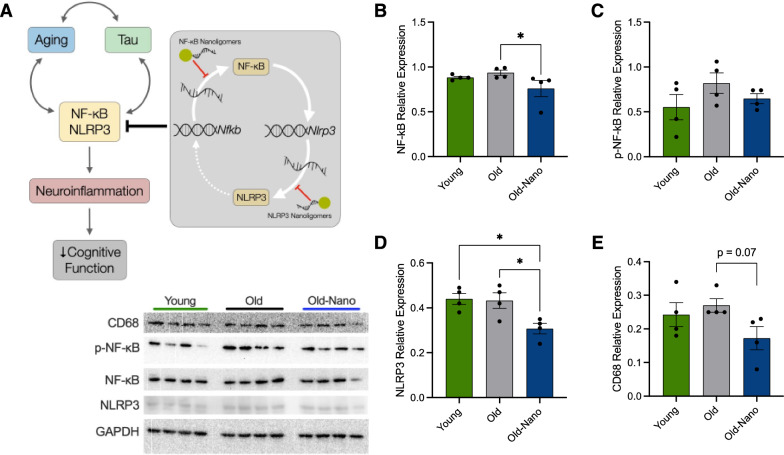
Short-term Nanoligomer treatment downregulates NF-κB and NLRP3 in the brain. **A** NF-κB and NLRP3 interact to drive inflammation, but Nanoligomers that block their translation may reduce age- and tau-associated neuroinflammation. **B**–**E** Western blot data showing protein levels of NF-κB, phosphorylated (p)-NF-κB, NLRP3 and Cluster of Differentiation 68 (CD68) in the hippocampus of young (8 months) and older (19 months) wildtype mice, and older mice 16 h after treatment with a NF-κB/NLRP3-targeting Nanoligomer cocktail. Raw immunoblots shown at bottom left and full blots shown in supplementary data file. Data analyzed via ordinary one-way ANOVA and Tukey’s post-hoc testing (p-NF-κB, F (2, 9) = 1.53; NLRP3, F (2, 9) = 6.89) or non-parametric one-way ANOVA for with Dunn’s post-hoc testing for non-normally distributed data (NF-κB, Kruskal–Wallis statistic = 7.47; CD68, Kruskal–Wallis statistic = 5.11). N = 4/group for all analyses; *p < 0.05

The transcription factor Nuclear Factor Kappa B (NF-κB) and inflammasome member NLR Family Pyrin Domain Containing 3 (NLRP3) are key drivers of age- and tau-associated neuroinflammation, and both play central roles in adverse processes that drive brain aging and AD (e.g., immune activation and pro-inflammatory signaling) [6, 7]. The roles of these proteins in brain aging and neurodegenerative disease are multifaceted, and NF-κB in particular may also be neuroprotective (e.g., by regulating synaptic plasticity and neurotrophic factors) [8]. However, a preponderance of recent studies have demonstrated that the inhibition of either NF-κB or NLRP3 alone reduces immune activation/pro-inflammatory cytokine release in the brain and improves cognitive function [9–11], suggesting that the NF-κB/NLRP3 signaling axis may be a promising therapeutic target. Despite these findings, current treatments that target NF-κB and NLRP3 may have off-target effects (e.g., by inhibiting activity of other enzymes) [12] and/or poor biodistribution, and many systemic anti-inflammatory treatments (e.g., immunosuppressive steroids) do not cross the blood–brain barrier. To address these concerns, our team recently developed a novel Nanoligomer (nucleic acid therapeutic) cocktail that targets both NF-κB and NLRP3 DNA, to inhibit transcription, and mRNA, to inhibit translation (Fig. 1A). Nanoligomers comprise an antisense peptide nucleic acid (PNA) conjugated to a gold nanoparticle for improved delivery [13–18], and this particular Nanoligomer cocktail was developed via in-depth screening studies for different targets to reduce neuroinflammation, in which NF-κB emerged as the top single target and NLRP3 showed a synergistic interaction [16, 19]. Our recent reports demonstrate that this NF-κB/NLRP3 Nanoligomer cocktail is highly specific, crosses the blood–brain barrier, is bioavailable across various brain regions [18, 20], downregulates its targets both at the RNA and protein level, and inhibits neuroinflammation in vitro and in vivo [15, 16, 18–20], yet its influence on cognitive function in the context of brain aging and tauopathy has not been thoroughly explored.

In the present study, we evaluated the efficacy of targeting both NF-κB and NLRP3 in brain aging and tauopathy by treating both old wildtype mice and transgenic rTg4510 mice (a model of tauopathy commonly used in neurodegeneration research) with our Nanoligomer cocktail. We found that this treatment: (1) improved cognitive function and broadly reduced neuroinflammation in both models; (2) reduced tau pathology and glial cell activation while conferring neuroprotection; (3) reversed transcriptome changes common to brain aging and tauopathy; and (4) exerted favorable systemic effects. Our results show that selective targeting of both NF-κB and NLRP3 is a potent strategy for reducing neuroinflammation, and they provide a basis for similar studies in larger animal models and/or pilot clinical trials.

## Results

### Short-term Nanoligomer treatment downregulates NF-κB and NLRP3 in the hippocampus

NF-κB and NLRP3 are central mediators of age-related inflammation (i.e., “inflammaging”), neuroinflammation, innate immune responses and cytokine release, including in the brain [6, 7, 21, 22]. Our published data show that NF-κB/NLRP3-targeting Nanoligomers downregulate these targets in multiple brain regions [18, 20]. However, to confirm this in the present study, we first treated older (aged 19 months) male and female C57Bl/6J mice with the same Nanoligomer cocktail or saline control (1 × intraperitoneal injection; 150 mg/kg body weight). Younger (aged 8 months) male and female mice, as a reference group, also received a saline injection. After 16 h, mice were euthanized and immunoblots were performed on isolated whole hippocampus. We observed significant reductions in both NF-κB and NLRP3 in old treated vs. untreated mice (Fig. 1B, D), and a modest but non-significant increase in phosphorylated (activated) NF-κB in older vs. young control animals that was absent in older treated animals (Fig. 1C). These effects of acute Nanoligomer administration on central mediators of neuroinflammation and innate immune responses are consistent with our reports showing that the drug crosses the blood–brain barrier and engages its targets [13, 14, 16, 18, 20], suggesting the potential for protective effects on mediators of neuroinflammation downstream of NF-κB/NLRP3 (e.g., reactive glia, pro-inflammatory gene induction and cytokine release) [23, 24]. Consistent with this idea, even with the short duration of this treatment, we also found a nearly significant trend for reduced expression of Cluster of Differentiation 68 (CD68), a marker of pro-inflammatory microglia in Nanoligomer-treated old mice (Fig. 1E).

### Long-term Nanoligomer treatment improves cognitive function in old wildtype and tauopathy mice

**Fig. 2:**
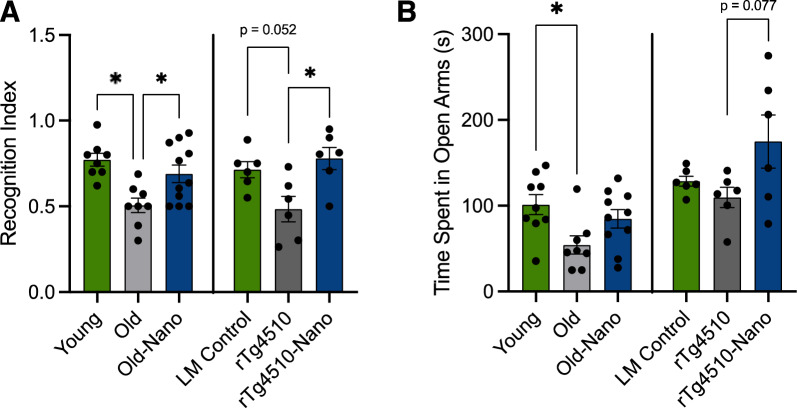
Long-term treatment with Nanoligomers targeting NF-κB and NLRP3 improves cognitive-behavioral function in old wildtype and tauopathy mice. **A** Novel Object Recognition index (short-term recognition memory) quantified as time exploring the novel object versus total object exploration time in young, old and old Nanoligomer-treated wildtype mice, as well as littermate (LM) controls, rTg4510 tauopathy and rTg4510 Nanoligomer-treated mice. **B** Time spent in open arms during Elevated Plus Maze testing, a reflection of anxiety-like behavior, in the same mice. Data analyzed via one-way ANOVA and group differences determined via Tukey’s post-hoc multiple comparison test. Novel Object Recognition: young and old mice, F (2, 24) = 7.61; rTg4510 mice, F (2, 15) = 6.03. Elevated plus maze: young and old mice, F (2, 24) = 4.31; rTg4510 mice, F (2, 15) = 2.97. N = 6–11/group, all data points shown; *p < 0.05

Although NF-κB and NLRP3 play complex roles in brain health [8], most data suggest that inhibiting these proteins individually improves cognitive function [25, 26]. Therefore, given our finding that acute Nanoligomer treatment reduced NF-κB and NLRP3 in the hippocampus (a key area of the brain involved in memory and cognitive function) and the central role of neuroinflammation in cognitive dysfunction with brain aging and AD [27], we next tested the hypothesis that long-term Nanoligomer treatment (150 mg/kg body weight; 3 × week for one month) would improve cognitive function in old C57Bl/6J male and female mice. In addition, because tauopathy (a key hallmark of AD) is associated with NF-κB/NLRP3 activation [28] and cognitive dysfunction [29], we also treated 2 month-old male and female rTg4510 mice, a common model of tauopathy based on expression of human P301L tau in the forebrain, using the same approach. Importantly, we did not note any adverse effects with this treatment, and all body weights remained stable. After the treatment, we measured short-term recognition memory via Novel Object Recognition testing and anxiety-like behavior via Elevated Plus Maze testing. In Novel Object Recognition testing, we found that treated older and tauopathy mice performed significantly better (as measured by recognition indices) than untreated controls, and similarly to younger mice and littermate controls, respectively (Fig. 2A). In the Elevated Plus Maze test, we observed a non-significant trend for treated older and tauopathy mice to spend more time in open maze arms than untreated mice, reflecting reduced anxiety (Fig. 2B). Finally, in tests of grip strength, which we measured because of its relationship to cognitive function and overall health [30], we found a slight but non-significant trend for improvements in old treated mice, although these effects were not noted in treated tauopathy mice, and among all animals we observed no differences in frailty, which is closely associated with cognitive decline [31] (Supplementary Figure S1). Future, more comprehensive studies are needed to assess how NF-κB/NLRP3-targeting Nanoligomer treatment affects other cognitive and peripheral functional outcomes (e.g., spatial memory, treadmill endurance, wire hang, rotarod performance). However, these results show that long-term Nanoligomer treatment improves several domains of cognitive-behavioral function in old, non-transgenic mice, and in tauopathy model mice, without overt adverse effects on neuromuscular function/health.

### Broad Nanoligomer-induced reductions in neuroinflammation correlate with cognitive function

**Fig. 3:**
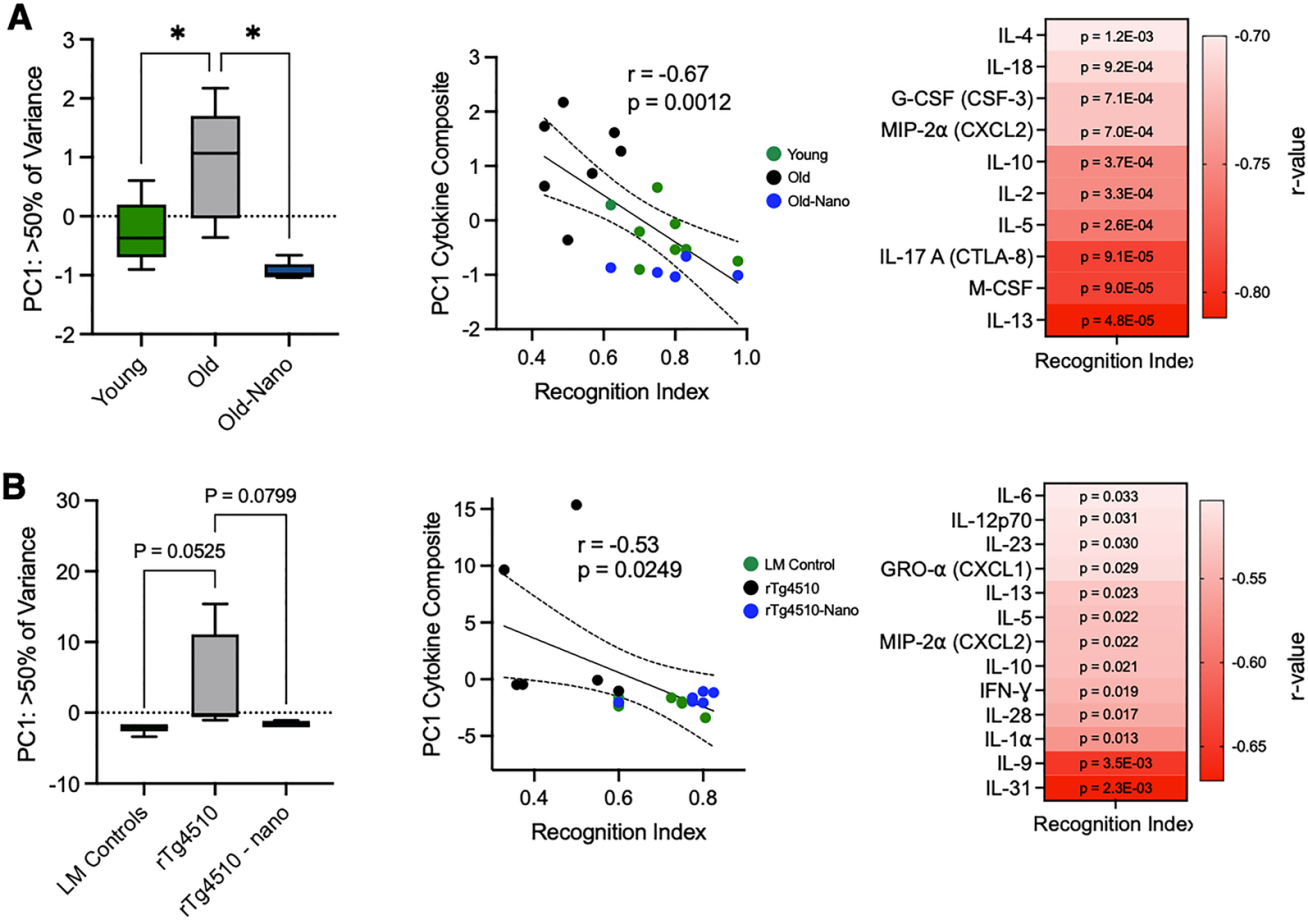
Broad reductions in neuroinflammation with Nanoligomer treatment are related to greater cognitive-behavioral function in old wildtype and tauopathy mice. **A** Principal Component 1 (PC1) from PC analysis of 36 cytokines in hippocampus of young, old and old Nanoligomer-treated wildtype mice (left panel). Correlation of PC1 values with Novel Object Recognition Index scores in the same mice (middle panel) and heatmap showing magnitude of individual cytokine correlations with Recognition Index (right panel). **B** The same PC and cytokine analyses in littermate (LM) controls, rTg4510 tauopathy and rTg4510 Nanoligomer-treated mice. PC group comparisons analyzed via one-way ANOVA and Tukey’s post-hoc multiple comparison test (young and old mice, F (2, 19) = 15.64; rTg4510 mice, F (2, 15) = 4.05). Correlations determined as simple Pearson correlation coefficients (r), and all analyses N = 6–11/group; *p < 0.05

The activation of NF-κB and NLRP3 via aging or tauopathy induces the production of many pro-inflammatory cytokines [32]. Therefore, we performed multiplex ELISAs on brains from the same mice (long-term Nanoligomer-treated) described above, to assess levels of key cytokines involved in neuroinflammation and innate immune responses. Principal component analyses (PCA) revealed that aging and tauopathy were associated with robust and broad increases in many cytokines, whereas cytokine levels in treated old and tauopathy animals were similar to those in young mice and littermate controls, respectively (Fig. 3A, B, left panels). At the individual cytokine level, several important pro-inflammatory and immune-activating cytokines that increased with aging and tauopathy included Interleukin 5 (IL-5) and Macrophage-Inflammatory Protein 2 (MIP-2α), but these effects were reduced with Nanoligomer treatment. Similar trends were noted for most Interleukins (ILs), Monocyte Chemoattractant Proteins (MCPs), and Interferons (IFNs) (Supplementary Figure S2). Additionally, we found that cytokine levels (global/PC-based and individual) were significantly inversely correlated with short-term recognition memory (Fig. 3A, B center and right panels), which is consistent with existing evidence that increased cytokine levels in the brain are associated with cognitive decline [33]. Together, these data show that long-term NF-κB/NLRP3-targeting Nanoligomer treatment broadly reduces neuroinflammation, including many cytokines that are directly modulated by NF-κB and NLRP3 [22], and that these changes may contribute to improvements in cognitive function in old wildtype and tauopathy model mice.

### Reductions in pathological tau with Nanoligomer treatment

**Fig. 4:**
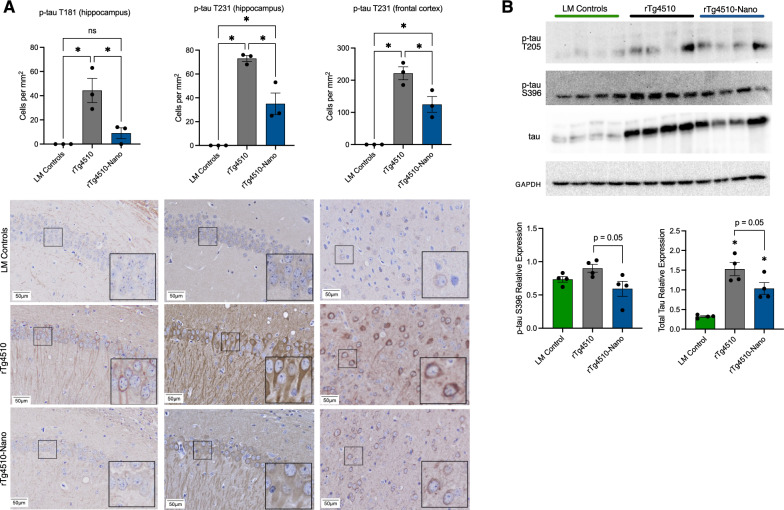
Reductions in pathological tau in the hippocampus with Nanoligomer treatment. **A** Quantification and representative immunohistochemistry images of phosphorylated tau (p-tau T181 and p-tau T231) in hippocampal and frontal cortex sections from littermate (LM) controls, rTg4510 tauopathy and rTg4510 Nanoligomer-treated mice. Insets in lower right corner of images are 2 × magnification of representative cell bodies indicated in smaller boxes. Note reduction in p-tau staining density with Nanoligomer treatment. **B** Raw immunoblots and quantification of total and phosphorylated tau species (p-tau T205 and p-tau S396) in the same mice (full blots shown in supplementary data file). Data analyzed via one-way ANOVA and group differences determined via Tukey’s post-hoc multiple comparison test (p-tau T181, F (2, 6) = 13.79; p-tau T231 hippocampus, F (2, 6) = 45.62; p-tau T231 cortex, F (2, 6) = 37.18; p-tau S396, F (2, 9) = 3.89; total tau, (F (2, 9) = 21.83). Immunohistochemistry, N = 3/group; immunoblots, N = 4/group; *p < 0.05

Importantly, tau pathology can both activate and be exacerbated by NF-κB and NLRP3 [34–36], driving cycles of inflammation-mediated tau propagation and toxicity [37], and phosphorylated (activated) tau is closely associated with cognitive dysfunction [38]. To characterize the effects of Nanoligomer treatment on tau, we performed immunohistochemistry for two tau residues (phosphorylated at threonine-181 and threonine-231) that have been associated with AD pathology and progression [39, 40]. We found that these tau species increased in hippocampus and cortex of rTg4510 mice, similar to what others have reported in tauopathy models [41, 42], and that this effect was attenuated with Nanoligomer treatment (Fig. 4A). We also performed immunoblots for tau, including for phosphorylated species more typical of later pathology, and we found that long-term Nanoligomer treatment was associated with modest reductions (p = 0.05) in both total and phosphorylated (at serine 396) tau using this approach (Fig. 4B).

**Fig. 5:**
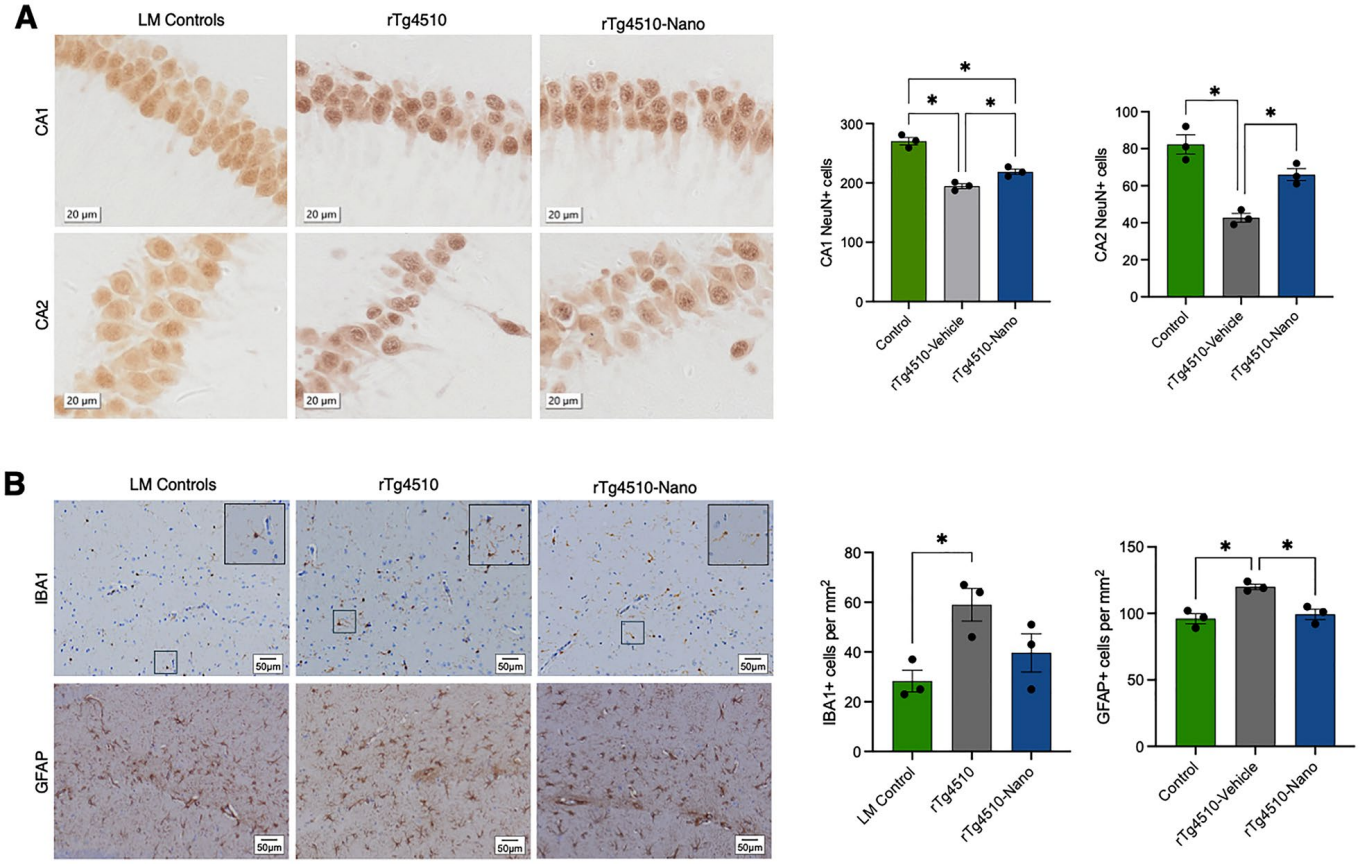
Changes in glial cell and neuronal numbers with Nanoligomer treatment in tauopathy mice. **A** Representative immunohistochemistry images (left) and counts (right) for cells positive for Fox-3 (NeuN) neuronal marker in CA1 and CA2 regions of the hippocampus in littermate (LM) controls, rTg4510 tauopathy and rTg4510 Nanoligomer-treated mice. **B** Immunohistochemistry images (left) and counts (right) for cells positive for Ionized Calcium-Binding Adapter molecule 1 (IBA1) and Glial Fibrillary Acidic Protein (GFAP) in the same mice. Data analyzed via one-way ANOVA and group differences determined via Tukey’s post-hoc multiple comparison test (CA1 NeuN, F (2, 6) = 57.69; CA2 NeuN, F (2, 6) = 27.59; IBA1, F (2, 6) = 5.95; GFAP, F (2, 6) = 15.16). N = 3/group; *p < 0.05

Finally, in these same mice, additional immunohistochemistry analyses showed that Nanoligomer treatment and the reduced tau burden we observed were associated with: (1) neuroprotection, as reflected by greater numbers of Fox-3 (NeuN)-positive cells in the CA1 and CA2 regions of the hippocampus (Fig. 5A); and (2) fewer potentially reactive glia, as reflected by reductions in Glial Fibrillary Acidic Protein (GFAP)-positive astrocytes and a non-significant trend for reductions in Ionized Calcium-Binding Adapter molecule 1 (IBA1)-positive microglia (Fig. 5B). Although GFAP and IBA1 counts do not reflect neuroinflammation per se, these results suggest that NF-κB/NLRP3-targeting Nanoligomer treatment may suppress tau pathology and confer neuroprotection, and that this effect may involve changes in glial cell-associated processes.

### Reversal of age-related glial cell activation with Nanoligomer treatment

**Fig. 6:**
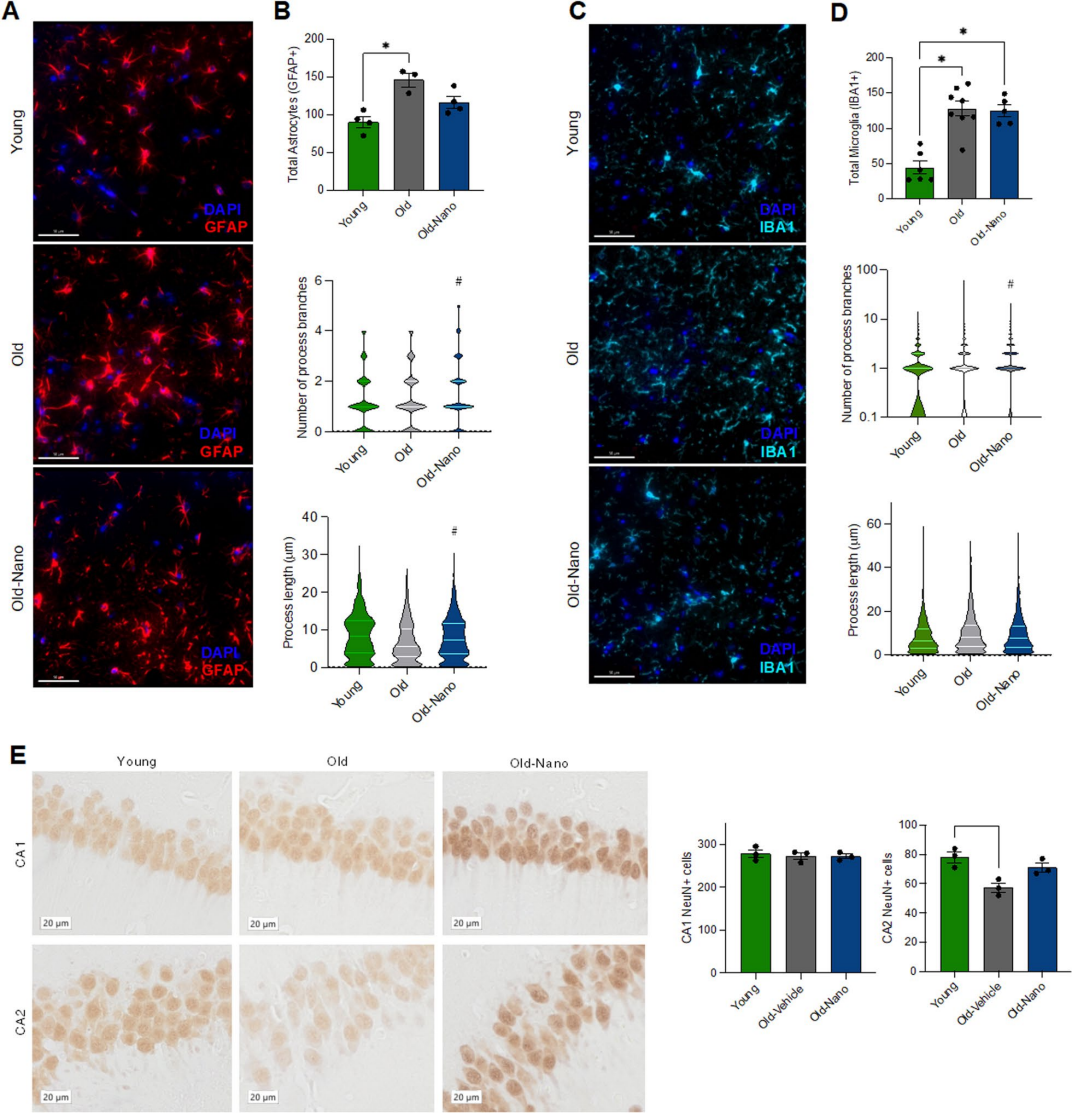
Reversal of age-related cellular changes in astrocytes and microglia with Nanoligomer treatment. **A** Immunofluorescence images of Glial Fibrillary Acidic Protein (GFAP) staining in hippocampus sections from young, old and old Nanoligomer-treated wildtype mice. Note general increase in GFAP/altered astrocyte morphology in old mice that is reversed with Nanoligomer treatment (scale bar 50 µm). **B** Quantification of GFAP + cells and process length and branch number. **C** Immunofluorescence images of Ionized Calcium-Binding Adapter molecule 1 (IBA1) staining in the same mice. Note general increase in IBA1/altered microglial morphology in old mice that is reversed with treatment (scale bar 50 µm). **D** Quantification of IBA1 + cells and process length and branch number. **E** Immunohistochemistry counts for Fox-3 (NeuN)-positive cells (neurons) in CA1 and CA2 regions of the hippocampus in the same mice. Data analyzed via one-way ANOVA and group differences determined via Tukey’s post-hoc multiple comparison test (GFAP, F (2, 8) = 11.64; IBA1, F (2, 16) = 22.10; CA1 NeuN, F (2, 6) = 0.20; CA2 NeuN, F (2, 6) = 9.81). *p < 0.05 vs. young; #p < 0.01 vs. old control

Although aged wildtype mice do not develop tau pathology, they do develop gliosis like humans. Indeed, in human aging/AD, glial cells (astrocytes and microglia) become immune-activated, secreting cytokines that impair neuronal and cognitive function [43]. This neuroinflammation is also associated with morphological changes [44, 45], including alterations in process length [46] and branching [47, 48], which have detrimental effects on neurons (e.g., synaptic dysfunction). To evaluate the effects of NF-κB/NLRP3-targeting Nanoligomer treatment on astrocyte and microglia activation/morphology with aging, we performed immunofluorescence staining on cortex tissue from old, old treated, and young mice. Consistent with previous findings showing that astrocyte activation increases with aging [44, 49], we found evidence of age-related increases in GFAP [50], but this effect was less pronounced in old treated mice (Fig. 6A). Similar to previous studies [51, 52], we also found that aging was associated with reduced astrocyte process length and branch number (Fig. 6B). However, these effects were reversed with Nanoligomer treatment. We also found age-related increases in the activation of microglia, as reflected by increases in IBA1 (Fig. 6C). This effect of aging on microglia was not altered by Nanoligomer treatment, although we did observe changes in microglial process length and branch number in old animals that were attenuated with treatment (Fig. 6D). Finally, similar to our observations in rTg4510 mice, using immunohistochemistry we found that all of these glial cell changes were associated with a reduction in NeuN + cells in old mice that was absent in treated animals, although only in the CA2 region of the hippocampus (Fig. 6E). Collectively, these results suggest that the beneficial effects of Nanoligomers targeting NF-κB/NLRP3 in old mice may be due in part to reducing age-related changes in glial cells and the preservation of neurons in the hippocampus.

### Transcriptome signatures of inflammation and reduced neuronal health are reversed with Nanoligomer treatment

**Fig. 7:**
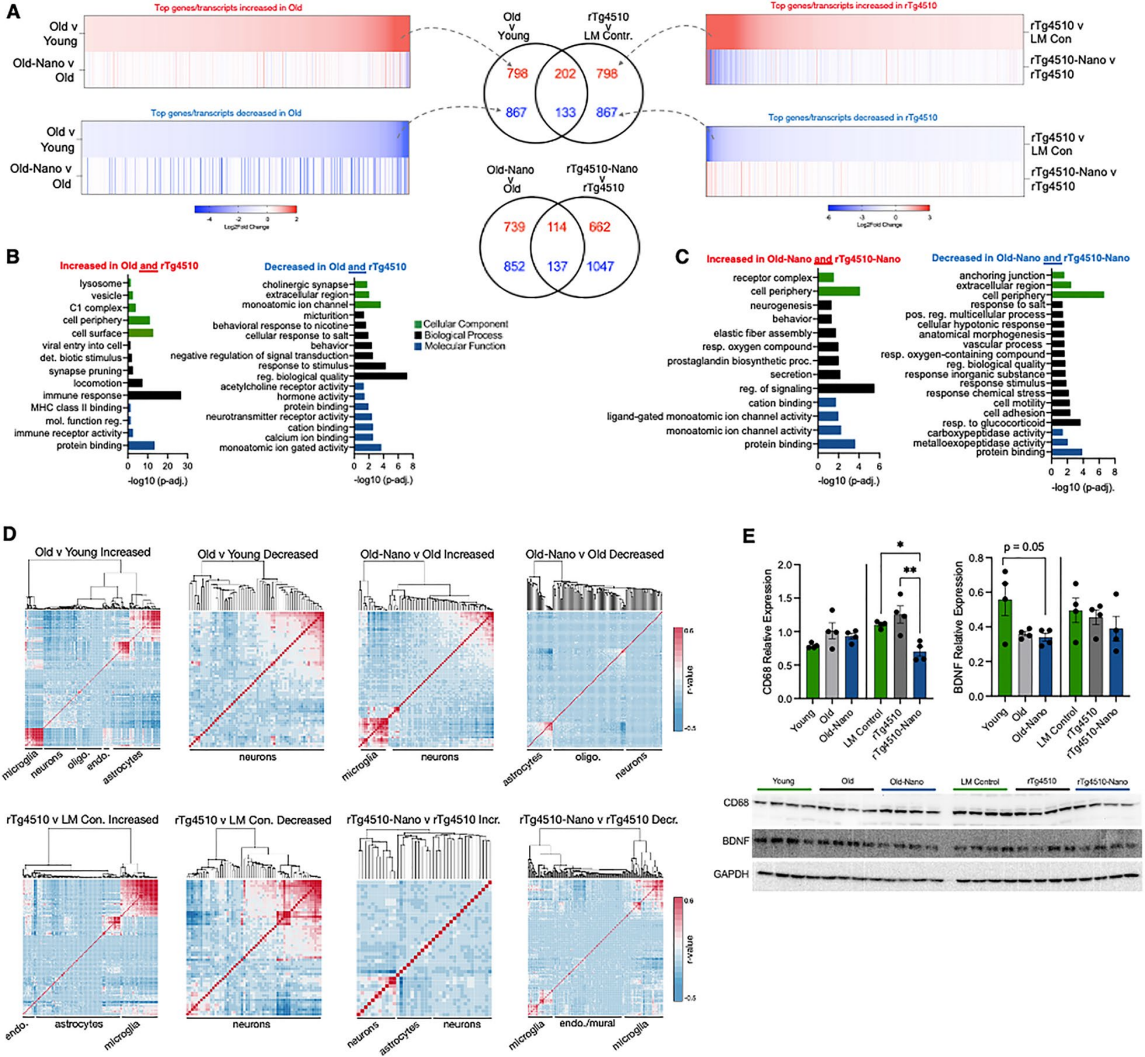
Transcriptome signatures of inflammation and reduced neuronal health with aging and tauopathy are reversed with Nanoligomer treatment. **A** Heatmaps showing Log2Fold differences for the top 1000 increased and decreased transcripts in old vs. young control mice and rTg4510 tauopathy mice vs. littermate (LM) controls (top heatmap rows), and Log2Fold differences for the same genes/transcripts in treated old and rTg4510 mice vs. their untreated counterparts (bottom heatmap rows). Venn diagrams in center show genes/transcripts that were increased or decreased in both old and rTg4510 mice, and genes/transcripts that were reversed in terms of relative expression in both treated old and treated rTg4510 mice. **B** Gene ontology analyses of genes/transcripts that were increased or decreased in both old and rTg4510 mice. **C** Gene ontology analyses of genes/transcripts that were increased or decreased in both treated old and treated rTg4510 mice. **D** Heatmaps showing hierarchical clustering and correlations between gene/transcript expression levels in bulk RNA-seq data from the present study and a single-cell RNA-seq dataset. Cell types associated with major gene expression clusters annotated; note glial cells associated with increased clusters and neurons associated with decreased clusters in both old and rTg4510 mice, and partial reversal of these patterns in heatmaps showing Nanoligomer treatment. **E** Quantifications and raw immunoblots for Cluster of Differentiation 68 (CD68) and Brain-Derived Neurotrophic Factor (BDNF) in animals from all groups (full blots shown in supplementary data file). Gene expression differences analyzed using DESeq2 algorithms and immunoblot data analyzed via one-way ANOVA with group differences determined via Tukey’s post-hoc multiple comparison test (CD68: young and old mice, F (2, 9) = 2.366; CD68, rTg4510 mice, F (2, 9) = 11.25; BDNF: young and old mice, F (2, 9) = 4.74; rTg4510 mice, F (2, 9) = 0.71). N = 4/group for all analyses; *p < 0.05

Perturbations in the transcriptome affect downstream biological processes that mediate brain aging/AD [53]. To provide broad insight into how Nanoligomer treatment affected genes/pathways most related to aging and tauopathy, we performed RNA-seq on the hippocampus. We found that aging and tauopathy were associated with gene expression changes (Fig. 7A, top rows of heatmaps) that were largely reversed in terms of relative expression with Nanoligomer treatment (Fig. 7A, bottom rows of heatmaps). For example, one of the top transcripts that increased with aging but decreased with Nanoligomer treatment was S100 calcium-binding protein A8 (*S100A8*), which activates NF-κB and induces microglial activation [54]. One of the top transcripts that increased with tauopathy but decreased with Nanoligomer treatment was Interleukin 17 Receptor E (*IL17RE*), which is also associated with the activation of NF-κB [55] and cognitive dysfunction [56].

Given the above observations and because aging and tau pathophysiology intersect [57], to understand how Nanoligomer treatment affected genes/pathways common to both aging and tauopathy, we compared the top 1000 most increased/decreased transcripts common to both aging and tauopathy, and those same transcripts in old and tauopathy-treated mice (Fig. 7A, top Venn diagrams). Gene ontology (GO) analyses revealed that increased genes common to both aging and tauopathy reflected pathways associated with inflammation, immune responses and cognitive dysfunction, including immune receptor activity, major histocompatibility complex (MHC) binding, and synapse pruning (Fig. 7B, left). Decreased genes/transcripts common to both aging and tauopathy comprised pathways associated with neurotransmitter receptor activity and signal transduction (Fig. 7B, right). However, these patterns were generally reversed with treatment. That is, among the transcripts that changed in both treated old and treated tauopathy mice (Fig. 7A, bottom Venn diagrams), those that were increased reflected pathways involved in brain aging and neuronal/cognitive function including ion channel activity, neuronal signaling, and neurogenesis (Fig. 7C, left), whereas common decreased transcripts with treatment in aged and tauopathy mice reflected pathways associated with inflammation and immune responses, including cell adhesion and stress response (Fig. 7C, right).

To determine which cell types might have contributed most to the observed transcriptome patterns, we performed a deconvolution analysis (intersecting bulk RNA-seq data with single-cell RNA-seq data to identify cell types most associated with gene expression differences in the bulk data [58]). This revealed a common pattern in both mouse/treatment models (Fig. 7D). With aging and tauopathy, increased gene expression signatures (i.e., of inflammation) were largely associated with glia, whereas decreased gene expression signatures were primarily associated with neurons—and in all cases these cell type-specific contributions to gene expression were at least somewhat reversed with Nanoligomer treatment. To confirm our RNA-seq data, we performed immunoblotting for several proteins whose gene expression levels changed (Figure S4), and we found similar patterns of protein expression for these targets. Also, given our findings above suggesting changes in glial cells and neuroprotection, we immunoblotted for CD68 (a marker of activated/reactive microglia [59]) and Brain-Derived Neurotrophic Factor (BDNF), a protein responsible for maintaining neuronal survival and growth [60] (Fig. 7E). We observed reduced CD68 in Nanoligomer-treated animals, although this effect was not significant in old mice, but no treatment-associated changes in BDNF in either model. Although not definitive, these results suggest that Nanoligomer targeting of NF-κB/NLRP3 favorably influences several important biological processes associated with brain aging and tauopathy, particularly those involved in neuroinflammation/immune activation and neuronal health, and that these effects may be mostly driven by changes in glial cells, upstream of neuroprotection (i.e., because the inflammation-related gene expression patterns that changed with treatment mapped to these cell types in deconvolution analyses, and neuron-intrinsic protective proteins like BDNF were unchanged).

### Favorable peripheral changes in response to Nanoligomer treatment

**Fig. 8:**
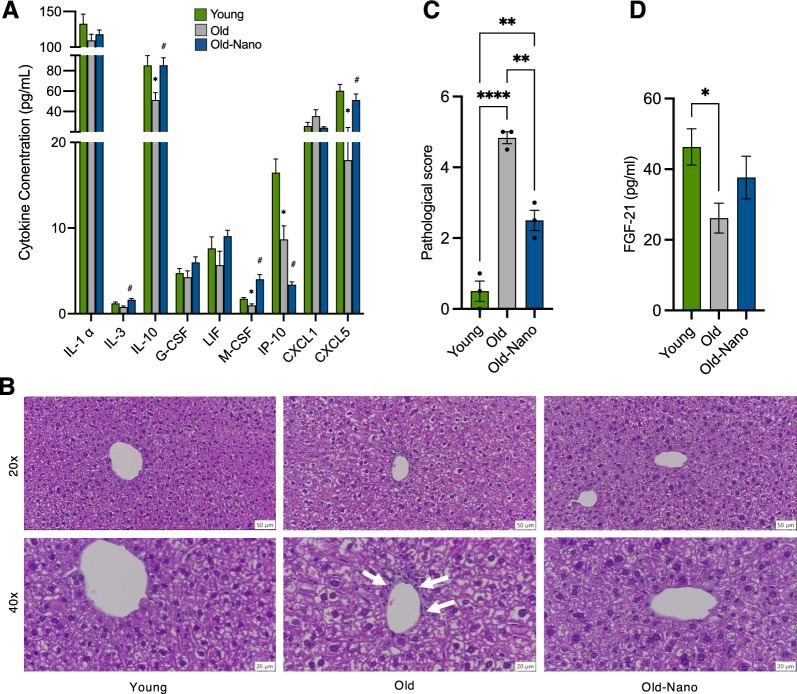
Favorable peripheral/systemic changes with long-term Nanoligomer treatment. **A** Cytokine concentrations in liver of young, old and old Nanoligomer-treated wildtype mice. N = 4/group; *p < 0.05 vs. young; ^#^p < 0.05 vs. old. **B** Representative immuno-histochemistry images showing infiltrating immune cells at portal vein border (white arrows) and **C** quantification of immune cell infiltration in the same mice. *p < 0.01. **D** Plasma levels of fibroblast growth factor 21 (FGF-21) measured by ELISA in the same mice. Data analyzed via one-way ANOVA with group differences determined via Tukey’s post-hoc multiple comparison test. Liver ELISAs: F (2, 23) = 1.44, 7.80, 5.54, 1.98, 2.00, 18.35, 29.98, 2.46 and 12.00; pathology scores: F (2, 6) = 72.57; plasma ELISA: F (2, 23) = 3.08. N = 6–11/group; *p < 0.01

Finally, current therapies meant to target neuroinflammation in the brain often have off-target effects [12, 61], and drug-induced hepatotoxicity is common following exposure to many bioactive compounds [62]. The liver is a key metabolic organ that processes such compounds and therefore a relevant target for therapies meant to help the brain [63]. To understand how Nanoligomer treatment affects the liver, we performed multiplex ELISAs on liver tissue from young, old, and old treated mice (Fig. 8A). We found that several pro-inflammatory and immune-activating cytokines that increased with aging were reduced with Nanoligomer treatment, including IFN-Ɣ inducible protein (IP-10) and chemokine ligand 1 (CXCL1). Additionally, the anti-inflammatory cytokine interleukin 10 (IL-10) was reduced with aging, but this effect was reversed with Nanoligomer treatment. We also found that aging was associated with evidence of hepatocyte polyploidy and polymorphonuclear leukocytes in the liver, a key source of tissue damage due to inflammation [64], and these effects were attenuated with Nanoligomer treatment (Fig. 8B, C). Furthermore, in Nanoligomer-treated old mice, these favorable changes in the liver were associated with a preservation of circulating (plasma) levels of fibroblast growth factor 21 (FGF-21), a hormone synthesized by the liver and linked with longevity and neuroprotection [65, 66]. Collectively, these results suggest that, in addition to their protective effects in the brain, Nanoligomers targeting NF-κB/NLRP3 are not hepatotoxic and may even favorably modulate several markers of inflammation and liver/peripheral health in old mice.

## Discussion

Neuroinflammation impairs cognitive function and is a major mechanism of brain aging and AD [5], both of which are linked with tau pathology. As such, there is an urgent need to identify safe and effective treatments that target neuroinflammation [67]. Age- and tau-related neuroinflammation is characterized by immune activation, glial cell (microglia and astrocytes) reactivity, and the release of neurotoxic, pro-inflammatory cytokines [68, 69], and many of these events involve signaling via NF-κB and NLRP3. These two proteins are upstream mediators of immune activation and key components of a “central axis” of neuroinflammation [7, 22, 70]. For example, NF-κB regulates many inflammatory processes, including the activation of pro-inflammatory cytokines and the transcription of neuroinflammatory genes [22], and NLRP3 is a major component of the inflammasome that directly regulates multiple immune response pathways (e.g., complement/cytokine signaling) [71]. Our key finding is that simultaneous, targeted downregulation of both NF-κB and NLRP3 via Nanoligomers reduces neuroinflammation and improves cognitive function in mouse models of both aging and tauopathy. This may be an important, clinically relevant finding, as these small molecule therapeutics are well positioned for further pre-clinical and eventual clinical translation.

Several recent investigations have shown that separately targeting either NF-κB or NLRP3 reduces neuroinflammation and improves cognitive function in multiple mouse models [9, 36, 72, 73]. For example, an NF-κB peptide inhibitor was shown to reduce inflammatory cytokine expression and microglial activation in 5xFAD mice [11], and inhibition of NLRP3 with the small molecule MCC950 is reported to reduce inflammation and improve cognitive function in APP/PS1 mice [9]. However, although NF-κB and NLRP3 signaling pathways are interconnected [21], no studies have investigated the effects of simultaneously targeting both of these proteins in old and/or tauopathy mice. To investigate this idea here, we performed an initial proof-of-concept study and found that short-term treatment with Nanoligomers targeting NF-κB and NLRP3 caused reductions in target proteins in the hippocampus of old mice, consistent with our previous reports on this same cocktail both in vitro [15] and in vivo [18]. Then, to provide translational relevance to this finding, we performed a longer intervention (proportional to that which might be conducted in older adults), in which we treated both old and tauopathy model mice with NF-κB/NLRP3-targeting Nanoligomers for one month. Notably, we found that this intervention was safe, well-tolerated, and elicited no adverse neuromuscular effects (e.g., on grip strength or frailty) or in the liver, a key metabolic organ (despite the potential protective role of NF-κB in various settings). We also found that this treatment improved short-term recognition memory and reduced anxiety, which is often co-morbid with dementia [74]. These results are important, because cognitive decline is a major risk factor for mild cognitive impairment and AD [75], and our findings are consistent with data from other studies on NF-κB and NLRP3 in mouse models of aging and AD [9, 11]. We note that intraperitoneal Nanoligomer delivery (the mode of treatment in our study) is a translational limitation, but our group is currently developing alternative approaches for administering these compounds, including via intranasal inhalation and oral routes. Demonstrating the efficacy of such approaches will be an important next step in this line of investigation.

Importantly, numerous pro-inflammatory/cytokine signaling pathways contribute to neuroinflammation-associated declines in cognitive function [5, 69, 76]. To address this in the present study, we probed the hippocampus for multiple cytokines that have been linked with cognitive dysfunction, brain aging and tauopathy. We found that NF-κB/NLRP3-targeting Nanoligomer treatment reduced almost all of these cytokines, with PCA showing a marked reversal of overall cytokine patterns in both aged and tauopathy mice. Moreover, these cytokine profiles were highly (inversely) correlated with cognitive-behavioral function in all animals studied. Collectively, these results suggest that the mechanism by which Nanoligomer treatment improves cognitive function involves broadly reducing neuroinflammation. This idea is in agreement with our previous studies showing that Nanoligomer treatment reduces neuroinflammation and improves cognitive function in prion-disease mice (a model of severe neurodegeneration) [18], and with numerous other studies showing that targeting neuroinflammation improves cognitive function in mouse models of accelerated brain aging [77]. In future work, it will be important to identify the specific NF-κB/NLRP3-associated cytokine pathways that impact cognitive function most directly, as some cytokines (e.g., IL-10) can have both protective [78] and deleterious effects in the brain [79].

Beyond specific cytokine pathways, neuroinflammation is linked with protein pathology (including tau accumulation) and glial cell dysregulation in aging and AD [4, 80–82]. However, the order of these events is incompletely understood, and neuro-inflammatory signaling mediators like NF-κB and NLRP3 have been reported to both contribute to and be activated by pathology [36]. In our study, we observed several effects of NF-κB/NLRP3-targeting Nanoligomers that are consistent with this idea. First, we found that reduced neuroinflammation with NF-κB/NLRP3-targeting Nanoligomers was associated with reductions in total and phosphorylated tau species representative of both early and later aging/AD pathology in rTg4510 mice [39, 40]. Because aggressive, transgene-driven expression of tau occurs in rTg4510 mice at young ages, our findings may suggest that the treatment protected against tau-induced NF-κB/NLRP3 activation that further promotes pathology in these animals. This idea would be in line with other studies showing that tau seeding can be reduced by small molecule inhibitors of NLRP3 [35], and that pharmacologically targeting neuroinflammation, in general, reduces the burden of phosphorylated tau [83, 84]. Second, in aging wildtype mice, we found that NF-κB/NLRP3 Nanoligomer-induced inhibition of neuroinflammation was associated with evidence of reduced glial cell activation. In contrast to rTg4510 and other transgenic/AD mouse models, aging wildtype mice do not develop tau or Aβ pathology. They do, however, develop glial cell-associated neuroinflammation, which has been linked with both age-related cognitive dysfunction and tau propagation [2, 5, 43, 69, 85]. Thus, our findings could suggest that NF-κB and NLRP3 also contribute to neuroinflammation regardless of pathology. Indeed, similar to previous studies showing that astrocytes and microglia become reactive/pro-inflammatory with aging [44, 49], here we found morphological and protein expression evidence of increased reactivity in both astrocytes and microglia of old mice—but NF-κB/NLRP3-targeting Nanoligomer treatment largely attenuated these effects. Together with our findings in tauopathy mice, these observations may suggest that NF-κB/NLRP3-associated neuroinflammation is a “proximal” target for enhancing cognitive function with aging and attenuating neurodegeneration (i.e., as depicted in Fig. 1). Future, more comprehensive studies using NF-κB/NLRP3-targeting Nanoligomers are needed to confirm the order of these events, and/or to evaluate the treatment’s effects on other neuroinflammation-associated pathologies (e.g., Aβ aggregation). Such work may be particularly important given the notable limitations of the rTg4510 mouse model used here. For example, recent data suggest that pathology in these animals may be driven in part by off-target effects of the tau transgene (e.g., disruption of other genes important to brain function) [86]. In our data, these genes were modestly differentially expressed (average Log2Fold differences ~ − 0.8) in transgenic animals, but their expression was not significantly altered with treatment and/or correlated with cognitive function metrics. This suggests that our overall findings may not have been driven by artifacts of the transgenic model but that the interpretation of our data should, perhaps, be taken with caution and it underscores the need for similar studies in additional transgenic models (e.g., 5xFAD, APP/PS1 or humanized hTau mice).

Finally, given the protective effects of NF-κB/NLRP3-targeting Nanoligomers in both old wildtype and tauopathy mice, along with evidence that aging and tauopathy may intersect [87], we used transcriptomics (RNA-seq) to provide insight into conserved mechanisms of action. We found common transcriptome signatures of aging and tauopathy reflecting differences in neuronal health, neuroinflammation and immune activation, similar to ours and others’ observations in pre-clinical models of brain aging [88, 89] and aging/AD in humans [90]. However, these age- and tauopathy-associated transcriptome effects were largely attenuated with Nanoligomer treatment, and even reversed in some cases. Deconvolution analyses of these data also showed that increased gene expression patterns with aging and tauopathy were mostly associated with astrocytes and microglia, whereas decreased gene expression patterns were mostly associated with neurons. NF-κB/NLRP3-targeting Nanoligomers at least partially reversed these patterns in both models, which is consistent with the idea that the treatment largely affected glial cells (i.e., because the direction of gene expression changes in these cells tracked with that of inflammatory gene expression). Given the important role of NF-κB/NLRP3 in other cell types, additional multi-omics and/or perhaps single-cell analyses would be helpful in confirming these observations and more thoroughly evaluating the up- and down-stream mechanisms by which targeting NF-κB/NLRP3 protects cognitive function.

## Conclusions and future directions

Our findings demonstrate that Nanoligomers specifically formulated to target NF-κB and NLRP3 are safe and well-tolerated, engage their target proteins in the brain, reduce neuroinflammation and improve cognitive function with both aging and tauopathy. Our molecular/transcriptomic data suggest overlapping mechanisms of action in these contexts, supporting the idea that NF-κB/NLRP3-associated neuroinflammation is a key target in aging and neurodegeneration. Future studies are needed to determine if this approach will work in larger animal models and/or humans, and to disentangle differential effects in short-term vs. long-term treatment periods with varying dosages.

## Methods

### Nanoligomer design and synthesis

Nanoligomers were synthesized by Sachi Bioworks as previously reported [13–18]. Briefly, Nanoligomers comprise an antisense peptide nucleic acid (PNA) conjugated to a gold nanoparticle for improved delivery and membrane transport. The PNA sequences, provided in [14, 15], were previously screened for solubility, self-complementarity, and off-targeting in the human genome. The PNA portions of the Nanoligomers (NLRP3 Sequence: CTTCTACTGCTCACAGG, NFKB Sequence: AGTGGTACCGTCTGCTA) were synthesized on a Vantage peptide synthesizer (AAPPTec, LLC) with solid-phase Fmoc chemistry. Fmoc-PNA monomers were obtained from PolyOrg Inc., with A, C, and G monomers protected with Bhoc groups. Following synthesis, the peptides were conjugated with gold nanoparticles and purified via size-exclusion filtration. Conjugation and concentration of the purified solution was monitored through measurement of absorbance at 260 nm (for detection of PNA) and 400 nm (for quantification of nanoparticles). The sequence of the synthesized PNA was confirmed using LC–MS [13].

### Animal husbandry

Older and younger male and female C57Bl/6J mice (aged 19 months and 8 months, respectively) were purchased from the National Institute on Aging aged rodent colony. Male and female rTg4510 mice and appropriate littermate controls (2 months of age) were purchased from the Jackson Laboratory (strain #024854). For the first part of the study (proof of concept to show that the Nanoligomer cocktail reduced both NF-κB and NLRP3 in the brain), cohorts were treated with Nanoligomer acutely (1 × intraperitoneal injection; 150 mg/kg body weight) and then studied 16 h later. For long-term treatment, mice were treated for 1 month (150 mg/kg body weight; 3 × week). Mice were housed throughout all experiments at ~ 18–23 °C on a reverse 12 light/12 dark cycle. Fresh water and *ad-libitum* food (Tekland 2918; 18% protein) was routinely provided to all cages. Animals were consistently health checked by the veterinary staff at Colorado State University.

### Animal sacrifice and tissue collection

Mice were culled in a fed state in the late morning. After deep anesthetizing with isoflurane, ~ 1 ml of blood was removed via cardiac puncture followed by cervical dislocation. The left hippocampus and a piece of left cortex tissue were removed, flash-frozen on dry ice, and stored at − 80 °C until further processing. The right hippocampus and right cortex were processed (paraffin-embedded) for immunostaining (details below).

### Immunoblotting

Frozen hippocampus samples were lysed in RIPA buffer containing 100 mM KCl, 40 mM Tris HCl, 10 mM Tris base, 5 mM MgCl_2_, 1 mM EDTA, and 1 mM ATP (pH 7.5), phoSTOP phosphatase inhibitors and cOmplete protease inhibitors (ThermoFisher). Sample protein concentration was determined using a BCA kit (ThermoFisher/Pierce), and 20 μg of protein per sample was separated by electrophoresis (Bio-Rad Criterion system), transferred to nitrocellulose membranes (Trans-Blot Turbo, Bio-Rad), and blocked in TBS-T with 5% Bovine Serum Albumin (BSA). Primary antibodies were purchased from ABclonal and included phosphorylated nuclear factor kappa B (p-NF-κB, 1:1000 dilution); nuclear factor kappa B (NF-κB, 1:1000); NLR Family Pyrin Domain Containing 3 (NLRP3, 1:1000); total tau (1:1000); p-tau S396 (1:1000); p-tau T205 (1:1000); Cluster of Differentiation 68 (CD68, 1:1000); Indoleamine 2,3‐dioxygenase 1 (IDO1, 1:1000); Interleukin 17 Receptor E (IL17RE, 1:1000); and Brain-Derived Neurotrophic Factor (BDNF, 1:1000). GAPDH (1:2000) was purchased from Novus Biologicals. Membranes were incubated in primary antibody (TBS-T in 5% BSA) for 24 h at 4°C, followed by HRP-conjugated secondary antibodies (TST-T in 5% milk; 1:5000; Cell Signaling) for 1 h. Membranes were then incubated in ECL, imaged on a ProteinSimple Fluor Chem imaging system, and quantified with ImageJ.

### Cognitive-behavioral and functional testing

Novel Object Recognition: For NOR testing, mice were assessed for short-term memory performance using no habituation phase [91]. Briefly, mice were placed in an opaque arena (50 cm × 50 cm) with two identical objects (tower of Legos) placed 8 cm from each side wall. After an inter-trial interval of two hours, mice were returned to the arena, but one of the old objects was replaced with a new object of similar shape and color (cell culture flask filled with multi-colored sand). Trials ended once 20 s total exploration time was reached, or 10 min had elapsed. If mice were unable to meet the exploration criteria of 20 s or 10 min, they were excluded from the analysis. Recognition index was calculated as novel object exploration time divided by 20 s [91].

Elevated Plus Maze: Elevated Plus Maze testing was performed as previously described [90, 92] and consisted of a cross with four arms (67 cm in length; 2 exposed arms, 2 enclosed arms). Mice were placed in the center of the maze facing an open arm and allowed to freely explore for 5 min. The amount of time in open arms (a measure of anxiety-like behavior) was recorded and analyzed with AnyMaze software.

Grip Strength: The grip strength test was performed as previously described [93]. Mice were tested using the DST-110 Digital Force Gauge (Maze Engineers). A total of 5 trials were performed per mouse and the highest and lowest values were discarded for analysis. The remaining 3 force values were averaged for each mouse. Data are presented as g force/g body weight.

Frailty Testing: Mouse frailty scores were assessed using an established scale [94]. Briefly, trained investigators blindly scored mice on a 31-point scale, which included measurements of health in the following areas: integumentary, physical/musculoskeletal, vestibular/auditory, ocular/nasal, digestive/urogenital, and respiratory systems. Higher scores indicate worse outcomes, while lower scores indicate better outcomes.

### Multiplexed ELISAs

Brain homogenates from all mice were assessed with a 36-plex Procartaplex cytokine/chemokine panel (ThermoFisher) as previously reported [15, 16], which included the following cytokines and chemokines: ENA-78 (CXCL5), Eotaxin (CCL11), GRO-α (CXCL1), IP-10 (CXCL10), MCP-1 (CCL2), MIP-1α (CCL3), MIP-1β (CCL4), MIP-2α (CXCL2), RANTES (CCL5), G-CSF (CSF-3), GM-CSF, IFN-α, IFN-γ, IL-1α, IL-1β, IL-2, IL-3, IL-4, IL-5, IL-6, IL-9, IL-10, IL-12p70, IL-13, IL-15/IL-15R, IL-17A (CTLA-8), IL-18, IL-22, IL-23, IL-27, IL-28, IL-31, LIF, MCP-3 (CCL7), M-CSF, and TNF-α. For systemic effects (Fig. 7), separate individual ELISAs were used. Briefly, 25uL of 10 mg/ml hippocampus, cortex, liver homogenate, or serum was processed using standard ELISA techniques and analyzed on a Luminex MAGPIX xMAP instrument. Standards for each cytokine/chemokine were used with 1:4 dilutions (eightfold dilutions) and background, controls and sample concentrations were determined from a standard curve using Five Parameter Logistic curve fit/quantification.

### Immunohistochemistry and immunofluorescence

Brain and liver tissues were fixed in 10% neutral buffered formalin at room temperature for at least 48 h. Brain samples were processed on a Leica TP1020 Automatic Benchtop Tissue Processor and embedded in paraffin wax (Cancer Diagnostics). For immunohistochemistry analyses of phosphorylated tau, paraffin-embedded tissue was sectioned at 4 µm, deparaffinized, rehydrated, sodium citate treated, and blocked in Tris A/2% donkey serum (Jackson ImmunoResearch), then incubated overnight in the following antibodies: phospho-tau (T181, 1:400 dilution; Invitrogen) and phospho-tau (T231, 1:400; ABClonal). Wash steps were performed using 2% bovine serum albumin and 2% Triton-X in 1 M TBS, and an ABC HRP peroxidase detection kit (Vector Laboratories) and ImmPACT DAB Substrate Peroxidase (HRP) Kit (Vector Laboratories) were used for a chromogen. Slides were counterstained with hematoxylin (ThermoFisher), secured with a coverslip in mounting medium and stored at room temperature until imaging. Whole tissue images were taken on an Olympus BX53 microscope with an Olympus DP70 camera using an Olympus UPlanSApo 20 × objective (N.A. = 0.75). Representative images were taken using an Olympus BX53 microscope with an Olympus DP70 camera using an Olympus UPlanFL N 40 × objective (N.A. = 0.75). To quantify and select the phosphorylation of tau-positive stained cells the manual threshold on the Count and Measure function of Olympus CellSens software (v1.18) was used. For immunofluorescence analyses of GFAP, IBA1 and glial cell process length and branching, sections were incubated overnight in primary antibodies: IBA1 (1:50 dilution; Abcam) and GFAP (1:100; Dako). Slides were washed with TBS and incubated for 1 h in the dark with AlexaFluor 555 or 647 (Invitrogen) secondary antibodies at 1:500 dilution and 2% normal donkey serum, then washed and incubated in Hoechst dye (1:2000; ThermoFisher) for 3 min. Slides were mounted with Prolong Gold Antifade mounting media (ThermoFisher) and stored in the dark at 4 °C prior to imaging. Images were captured using an Olympus BX63 fluorescence microscope equipped with a motorized stage and Hamamatsu ORCA-flash 4.0 LT CCD camera using a 40 × Olympus X-Apochromat air objective (N.A. = 0.80). For astrocyte/microglial process length and number analyses, four regions between the dentate gyrus and CA1-CA3 region of the hippocampus were imaged at 40×. To identify/quantify processes and branches, skeletonization of GFAP+ astrocytes and IBA1+ microglia was performed using IMARIS software as previously described [95] to trace and quantify stained filaments. For histology analyses of liver, tissues were sectioned on a ThermoFisher HM 325–2 microtome at 5 μm thickness and mounted on positively charged glass slides (Superfrost Plus, Cancer Diagnostics). Liver sections were deparaffinized and stained with hematoxylin and eosin (Cancer Diagnostics), and whole tissue images were taken for analysis using an Olympus DP70 camera and an Olympus UPlanSApo 20 × objective. Livers were scored for pathological infiltration of immune cells specifically around the portal triad, and for overall hepatocyte morphology by a trained animal pathologist.

### RNA sequencing and transcriptomic analyses

Transcriptome (RNA-seq) and gene expression analyses were performed on whole hippocampus via methods previously described [89, 90]. Frozen samples (n = 6/group; 3M and 3F) were transferred into Trizol (ThermoFisher) and homogenized on ice (SP Bel-Art ProCulture Micro-Tube Homogenizer System; VWR Scientific). RNA was isolated using an RNA-specific spin column kit (Direct-Zol, Zymo Research) and treated with DNase to remove genomic DNA. Poly(A)-selected libraries were generated using magnetic oligo dT beads (ThermoFisher) and Illumina TruSeq kits. Libraries were sequenced on an Illumina NovaSeq 6000 platform to yield > 40 M 151-bp paired-end FASTQ reads/sample. Reads were trimmed and filtered with the fastp program [96], then aligned to the mm10 *Mus musculus* genome using the STAR aligner [97]. Differential gene expression was analyzed with DESeq2 [98] and genes/transcripts with BaseMean read counts of < 10 were filtered and removed for analyses [99, 100]. Differentially expressed genes were analyzed for gene ontology (GO) enrichment using the Metascape program [101], and heatmaps were constructed with GraphPad Prism software. Deconvolution analyses of bulk RNA-seq data were performed as previously described [88] using a published protocol and R script [58]. Briefly, single-cell RNA-seq data on mouse hippocampus (SMART-seq, 2019; 10x-SMART-seq taxonomy, 2020) were downloaded from the Allen Brain Map portal [102], and cells from the hippocampus, including astrocytes, endothelial cells, microglia, oligodendrocytes and neurons, were sub-setted for analysis. Normalized DESeq2 gene counts for old vs. young mice, old Nanoligomer-treated vs. old, rTg4510 vs. LM Controls, and rTg4510 Nanoligomer-treated vs. rTg4510 comparisons in bulk RNA-seq data were filtered for the top 250 genes by p-value, and then sorted by Log2Fold change. Sorted gene lists were scaled to the single-cell RNA-seq dataset, normalized expression of the same genes in the single-cell dataset was obtained and used for dimensional reduction via principal component analysis and hierarchical clustering/heat mapping to obtain cell-type specific contributions to differences in the bulk RNA-seq comparisons, and cell types associated with each cluster were determined using CellMarker 2.0 [103].

### Statistics

One-way ANOVA with Tukey’s *post-hoc* testing was used to assess differences among groups in protein/cytokine levels and measures of cognitive function, as well as glial cell morphology, and simple linear regression (Pearson correlations) was used to relate cytokine levels to cognitive function data. A non-parametric ANOVA equivalent (Kruskal–Wallis test) was used for small sample sizes with non-normally distributed data. All data were analyzed and presented using GraphPad Prism software. Differentially expressed genes were detected using DESeq2, and genes were sorted by p-value and Log2Fold change to construct volcano plots and heatmaps, respectively. The Metascape program was used for GO and TRRUST analyses [101].

### Supplementary Information

Additional file 1:** Figure S1.** Nanoligomers targeting NF-κB and NLRP3 increase grip strength in old wildtype mice and have no adverse effects on overall physical health/frailty. Left: Forelimb grip strength in young, old and old Nanoligomer-treated wildtype mice, as well as littermate (LM) controls, rTg4510 tauopathy and rTg4510 Nanoligomer-treated mice. Right: Frailty Index in the same animals. N = 6–11/group; *p < 0.05. **Figure S2.** Nanoligomers targeting NF-κB and NLRP3 reverse age- and tauopathy-related increases in many cytokines in the brain. Multiplex ELISA analyses of prefrontal cortex tissue from young, old and old Nanoligomer-treated wildtype mice, as well as littermate (LM) controls, rTg4510 tauopathy and rTg4510 Nanoligomer-treated mice. N = 6–11/group; *p < 0.05 vs. young/LM; ^#^p < 0.05 vs. old/rTg4510; red lines represent limit of detection. **Figure S3.** Nanoligomers targeting NF-κB and NLRP3 modulate glial cell morphology in old mice. Representative images from Fig. 5 (left in each panel) and corresponding images (right in each panel) showing skeletonization (white lines) for assessing morphology in **A** astrocytes stained for GFAP and **B** microglia stained for IBA1. **Figure S4.** Immunoblotting confirmation of hits in RNA-seq data. Raw immunoblots and quantifications showing protein levels that track with RNA-seq gene expression levels for Indoleamine 2,3‐dioxygenase 1 (IDO1) and Interleukin 17 Receptor E (IL17RE), both of which have also been linked with differences in cognitive function, in young, old and old Nanoligomer-treated wildtype mice, as well as littermate (LM) controls, rTg4510 tauopathy and rTg4510 Nanoligomer-treated mice. *p < 0.05; **p < 0.01. Note: same blot/GAPDH loading controls as in Fig. 7.

Additional file 2.

## Data Availability

The RNA-seq data generated in this study are available on the Gene Expression Omnibus website under accession number GSE251888. All additional data are included in the data supplement, and raw data will be made available upon request to the corresponding author (T.J.L).

## References

[1] Xia X, et al. Aging and Alzheimer’s disease: comparison and associations from molecular to system level. Aging Cell. 2018;17(5): e12802. 29963744 10.1111/acel.12802 PMC6156542

[2] Wyss-Coray T. Ageing, neurodegeneration and brain rejuvenation. Nature. 2016;539(7628):180–6. 27830812 10.1038/nature20411 PMC5172605

[3] Heneka MT, Kummer MP, Latz E. Innate immune activation in neurodegenerative disease. Nat Rev Immunol. 2014;14(7):463–77. 24962261 10.1038/nri3705

[4] Metcalfe MJ, Figueiredo-Pereira ME. Relationship between tau pathology and neuroinflammation in Alzheimer’s disease. Mt Sinai J Med. 2010;77(1):50–8. 20101714 10.1002/msj.20163 PMC2904237

[5] Andronie-Cioara FL, et al. Molecular mechanisms of neuroinflammation in Aging and Alzheimer’s disease progression. Int J Mol Sci. 2023;24(3):1869. 36768235 10.3390/ijms24031869 PMC9915182

[6] Youm YH, et al. Canonical Nlrp3 inflammasome links systemic low-grade inflammation to functional decline in aging. Cell Metab. 2013;18(4):519–32. 24093676 10.1016/j.cmet.2013.09.010 PMC4017327

[7] Walker KA, et al. Connecting aging biology and inflammation in the omics era. J Clin Invest. 2022;132(14):158448. 10.1172/JCI158448 PMC9282936 35838044

[8] Mattson MP, Meffert MK. Roles for NF-κB in nerve cell survival, plasticity, and disease. Cell Death Differ. 2006;13(5):852–60. 16397579 10.1038/sj.cdd.4401837

[9] Dempsey C, et al. Inhibiting the NLRP3 inflammasome with MCC950 promotes non-phlogistic clearance of amyloid-β and cognitive function in APP/PS1 mice. Brain Behav Immun. 2017;61:306–16. 28003153 10.1016/j.bbi.2016.12.014

[10] Jha NK, et al. Nuclear factor-kappa β as a therapeutic target for Alzheimer’s disease. J Neurochem. 2019;150(2):113–37. 30802950 10.1111/jnc.14687

[11] Lindsay A, Hickman D, Srinivasan M. A nuclear factor-kappa B inhibiting peptide suppresses innate immune receptors and gliosis in a transgenic mouse model of Alzheimer’s disease. Biomed Pharmacother. 2021;138: 111405. 33756153 10.1016/j.biopha.2021.111405

[12] Kennedy CR, et al. A probe for NLRP3 inflammasome inhibitor MCC950 identifies carbonic anhydrase 2 as a novel target. ACS Chem Biol. 2021;16(6):982–90. 34003636 10.1021/acschembio.1c00218 PMC8218299

[13] McCollum CR, et al. Safety and biodistribution of nanoligomers targeting the SARS-CoV-2 genome for the treatment of COVID-19. ACS Biomater Sci Eng. 2023;9(3):1656–71. 36853144 10.1021/acsbiomaterials.2c00669

[14] McCollum CR, et al. Nanoligomers targeting human miRNA for the treatment of severe COVID-19 are safe and nontoxic in mice. ACS Biomater Sci Eng. 2022;8(7):3087–106. 35729709 10.1021/acsbiomaterials.2c00510

[15] Courtney CM, et al. Reversing radiation-induced immunosuppression using a new therapeutic modality. Life Sci Space Res. 2022;35:127–39. 10.1016/j.lssr.2022.05.002 36336358

[16] Sharma S, et al. Identifying an optimal neuroinflammation treatment using a nanoligomer discovery engine. ACS Chem Neurosci. 2022;13(23):3247–56. 36410860 10.1021/acschemneuro.2c00365

[17] McDonald JT, et al. Role of miR-2392 in driving SARS-CoV-2 infection. Cell Rep. 2021;37(3): 109839. 34624208 10.1016/j.celrep.2021.109839 PMC8481092

[18] Risen SJ, et al. Targeting neuroinflammation by pharmacologic downregulation of inflammatory pathways is neuroprotective in protein misfolding disorders. ACS Chem Neurosci. 2024;15(7):1533–47. 38507813 10.1021/acschemneuro.3c00846

[19] Sharma S, et al. Targeted-neuroinflammation mitigation using inflammasome-inhibiting nanoligomers is therapeutic in an experimental autoimmune encephalomyelitis mouse model. ACS Chem Neurosci. 2024;15(7):1596–608. 38526238 10.1021/acschemneuro.4c00024

[20] Risen S, et al. Large- and small-animal studies of safety, pharmacokinetics, and biodistribution of inflammasome-targeting nanoligomer in the brain and other target organs. ACS Pharmacol Transl Sci. 2024. 10.1021/acsptsci.4c00068. 10.1021/acsptsci.4c00068 PMC11555505 39539269

[21] Bauernfeind FG, et al. Cutting edge: NF-kappaB activating pattern recognition and cytokine receptors license NLRP3 inflammasome activation by regulating NLRP3 expression. J Immunol. 2009;183(2):787–91. 19570822 10.4049/jimmunol.0901363 PMC2824855

[22] Liu T, et al. NF-κB signaling in inflammation. Signal Transduct Target Ther. 2017;2:17023. 29158945 10.1038/sigtrans.2017.23 PMC5661633

[23] Shih RH, Wang CY, Yang CM. NF-kappaB signaling pathways in neurological inflammation: a mini review. Front Mol Neurosci. 2015;8:77. 26733801 10.3389/fnmol.2015.00077 PMC4683208

[24] Gao C, et al. Microglia in neurodegenerative diseases: mechanism and potential therapeutic targets. Signal Transduct Target Ther. 2023;8(1):359. 37735487 10.1038/s41392-023-01588-0 PMC10514343

[25] Datusalia AK, Sharma SS. NF-κB inhibition resolves cognitive deficits in experimental type 2 diabetes mellitus through CREB and glutamate/GABA neurotransmitters pathway. Curr Neurovasc Res. 2016;13(1):22–32. 26517200 10.2174/1567202612666151030104810

[26] Saggu R, et al. Astroglial NF-kB contributes to white matter damage and cognitive impairment in a mouse model of vascular dementia. Acta Neuropathol Commun. 2016;4(1):76. 27487766 10.1186/s40478-016-0350-3 PMC4973061

[27] Bettio LEB, Rajendran L, Gil-Mohapel J. The effects of aging in the hippocampus and cognitive decline. Neurosci Biobehav Rev. 2017;79:66–86. 28476525 10.1016/j.neubiorev.2017.04.030

[28] Jiang S, et al. Proteopathic tau primes and activates interleukin-1β via myeloid-cell-specific MyD88- and NLRP3-ASC-inflammasome pathway. Cell Rep. 2021;36(12): 109720. 34551296 10.1016/j.celrep.2021.109720 PMC8491766

[29] Di J, et al. Abnormal tau induces cognitive impairment through two different mechanisms: synaptic dysfunction and neuronal loss. Sci Rep. 2016;6(1):20833. 26888634 10.1038/srep20833 PMC4757872

[30] Jiang R, et al. Associations between grip strength, brain structure, and mental health in > 40,000 participants from the UK Biobank. BMC Med. 2022;20(1):286. 36076200 10.1186/s12916-022-02490-2 PMC9461129

[31] Fabrício DM, Chagas MHN, Diniz BS. Frailty and cognitive decline. Transl Res. 2020;221:58–64. 32045578 10.1016/j.trsl.2020.01.002

[32] Liu T, et al. NF-κB signaling in inflammation. Signal Transduct Target Ther. 2017;2(1):17023. 29158945 10.1038/sigtrans.2017.23 PMC5661633

[33] Flores-Aguilar L, et al. Cognitive and brain cytokine profile of non-demented individuals with cerebral amyloid-beta deposition. J Neuroinflammation. 2021;18(1):147. 34218796 10.1186/s12974-021-02169-0 PMC8254948

[34] Bhaskar K, et al. Pathological tau activates nuclear factor-kappa B (NF-κB) and drives neuroinflammation. Alzheimers Dement. 2022;18(S4): e069386. 10.1002/alz.069386

[35] Stancu IC, et al. Aggregated Tau activates NLRP3-ASC inflammasome exacerbating exogenously seeded and non-exogenously seeded Tau pathology in vivo. Acta Neuropathol. 2019;137(4):599–617. 30721409 10.1007/s00401-018-01957-y PMC6426830

[36] Van Zeller M, et al. NLRP3 inflammasome: a starring role in amyloid-beta- and tau-driven pathological events in Alzheimer’s disease. J Alzheimers Dis. 2021;83(3):939–61. 34366341 10.3233/JAD-210268 PMC8543248

[37] Wang C, et al. Microglial NF-κB drives tau spreading and toxicity in a mouse model of tauopathy. Nat Commun. 2022;13(1):1969. 35413950 10.1038/s41467-022-29552-6 PMC9005658

[38] Sydow A, et al. Tau-induced defects in synaptic plasticity, learning, and memory are reversible in transgenic mice after switching off the toxic Tau mutant. J Neurosci. 2011;31(7):2511–25. 21325519 10.1523/JNEUROSCI.5245-10.2011 PMC6623704

[39] Suárez-Calvet M, et al. Novel tau biomarkers phosphorylated at T181, T217 or T231 rise in the initial stages of the preclinical Alzheimer’s continuum when only subtle changes in Aβ pathology are detected. EMBO Mol Med. 2020;12(12): e12921. 33169916 10.15252/emmm.202012921 PMC7721364

[40] Lilek J, et al. Accumulation of pTau231 at the postsynaptic density in early Alzheimer’s disease. J Alzheimers Dis. 2023;92(1):241–60. 36744338 10.3233/JAD-220848 PMC10041451

[41] Song L, et al. Analysis of tau post-translational modifications in rTg4510 mice, a model of tau pathology. Mol Neurodegener. 2015;10:14. 25881209 10.1186/s13024-015-0011-1 PMC4391670

[42] Bennett RE, et al. Enhanced Tau aggregation in the presence of amyloid β. Am J Pathol. 2017;187(7):1601–12. 28500862 10.1016/j.ajpath.2017.03.011 PMC5500829

[43] Yu Y, et al. The role of glial cells in synaptic dysfunction: insights into Alzheimer’s disease mechanisms. Aging Dis. 2023. 10.14336/AD.2023.0718. 37548934 10.14336/AD.2023.0718 PMC10917533

[44] Deng Q, et al. Microglia and astrocytes in Alzheimer’s disease: significance and summary of recent advances. Aging Dis. 2023. 10.14336/AD.2023.0907. 37815901 10.14336/AD.2023.0907 PMC11272214

[45] Niraula A, Sheridan JF, Godbout JP. Microglia priming with aging and stress. Neuropsychopharmacology. 2017;42(1):318–33. 27604565 10.1038/npp.2016.185 PMC5143497

[46] Streit WJ, Walter SA, Pennell NA. Reactive microgliosis. Prog Neurobiol. 1999;57(6):563–81. 10221782 10.1016/S0301-0082(98)00069-0

[47] Rodríguez-Callejas JD, Fuchs E, Perez-Cruz C. Atrophic astrocytes in aged marmosets present tau hyperphosphorylation, RNA oxidation, and DNA fragmentation. Neurobiol Aging. 2023;129:121–36. 37302213 10.1016/j.neurobiolaging.2023.04.010

[48] Godeanu S, et al. Microglial morphology in the somatosensory cortex across lifespan. A quantitative study. Dev Dyn. 2023;252(8):1113–29. 36883224 10.1002/dvdy.582

[49] Clarke LE, et al. Normal aging induces A1-like astrocyte reactivity. Proc Natl Acad Sci USA. 2018;115(8):E1896–905. 29437957 10.1073/pnas.1800165115 PMC5828643

[50] Liddelow SA, Barres BA. Reactive astrocytes: production, function, and therapeutic potential. Immunity. 2017;46(6):957–67. 28636962 10.1016/j.immuni.2017.06.006

[51] Bondi H, et al. Complex and regional-specific changes in the morphological complexity of GFAP+ astrocytes in middle-aged mice. Neurobiol Aging. 2021;100:59–71. 33493951 10.1016/j.neurobiolaging.2020.12.018

[52] Popov A, et al. Astrocyte dystrophy in ageing brain parallels impaired synaptic plasticity. Aging Cell. 2021;20(3): e13334. 33675569 10.1111/acel.13334 PMC7963330

[53] Ham S, Lee SV. Advances in transcriptome analysis of human brain aging. Exp Mol Med. 2020;52(11):1787–97. 33244150 10.1038/s12276-020-00522-6 PMC8080664

[54] Wu M, et al. S100A8/A9 induces microglia activation and promotes the apoptosis of oligodendrocyte precursor cells by activating the NF-κB signaling pathway. Brain Res Bull. 2018;143:234–45. 30266587 10.1016/j.brainresbull.2018.09.014

[55] Liu Y, et al. Activation of the IL-17/TRAF6/NF-κB pathway is implicated in Aβ-induced neurotoxicity. BMC Neurosci. 2023;24(1):14. 36823558 10.1186/s12868-023-00782-8 PMC9951515

[56] Brigas HC, et al. IL-17 triggers the onset of cognitive and synaptic deficits in early stages of Alzheimer’s disease. Cell Rep. 2021;36(9): 109574. 34469732 10.1016/j.celrep.2021.109574

[57] Kovacs GG. Chapter 25—tauopathies. In: Kovacs GG, Alafuzoff I, editors. Handbook of clinical neurology. Amsterdam: Elsevier; 2018. p. 355–68.

[58] Marquez-Galera A, de la Prida LM, Lopez-Atalaya JP. A protocol to extract cell-type-specific signatures from differentially expressed genes in bulk-tissue RNA-seq. STAR Protoc. 2022;3(1): 101121. 35118429 10.1016/j.xpro.2022.101121 PMC8792262

[59] Jurga AM, Paleczna M, Kuter KZ. Overview of general and discriminating markers of differential microglia phenotypes. Front Cell Neurosci. 2020;14:198. 32848611 10.3389/fncel.2020.00198 PMC7424058

[60] Bathina S, Das UN. Brain-derived neurotrophic factor and its clinical implications. Arch Med Sci. 2015;11(6):1164–78. 26788077 10.5114/aoms.2015.56342 PMC4697050

[61] Sivamaruthi BS, et al. NF-κB pathway and its inhibitors: a promising frontier in the management of Alzheimer’s disease. Biomedicines. 2023;11(9):2587. 37761028 10.3390/biomedicines11092587 PMC10526355

[62] Fisher K, Vuppalanchi R, Saxena R. Drug-induced liver injury. Arch Pathol Lab Med. 2015;139(7):876–87. 26125428 10.5858/arpa.2014-0214-RA

[63] Hunt NJ, et al. Targeting the liver in dementia and cognitive impairment: dietary macronutrients and diabetic therapeutics. Adv Drug Deliv Rev. 2022;190: 114537. 36115494 10.1016/j.addr.2022.114537 PMC10125004

[64] Baggiolini M, et al. The polymorphonuclear leukocyte. Agents Actions. 1978;8(1–2):3–10. 345782 10.1007/BF01972395

[65] Hill CM, et al. FGF21 is required for protein restriction to extend lifespan and improve metabolic health in male mice. Nat Commun. 2022;13(1):1897. 35393401 10.1038/s41467-022-29499-8 PMC8991228

[66] Zhang Y, et al. The starvation hormone, fibroblast growth factor-21, extends lifespan in mice. Elife. 2012;1: e00065. 23066506 10.7554/eLife.00065 PMC3466591

[67] Wahl D, Cavalier AN, LaRocca TJ. Novel strategies for healthy brain aging. Exerc Sport Sci Rev. 2021;49(2):115–25. 33739944 10.1249/JES.0000000000000242 PMC7967995

[68] Carson MJ, Thrash JC, Walter B. The cellular response in neuroinflammation: the role of leukocytes, microglia and astrocytes in neuronal death and survival. Clin Neurosci Res. 2006;6(5):237–45. 19169437 10.1016/j.cnr.2006.09.004 PMC2630233

[69] Heneka MT, et al. Neuroinflammation in Alzheimer’s disease. Lancet Neurol. 2015;14(4):388–405. 25792098 10.1016/S1474-4422(15)70016-5 PMC5909703

[70] Afonina IS, et al. Limiting inflammation-the negative regulation of NF-κB and the NLRP3 inflammasome. Nat Immunol. 2017;18(8):861–9. 28722711 10.1038/ni.3772

[71] Kelley N, et al. The NLRP3 inflammasome: an overview of mechanisms of activation and regulation. Int J Mol Sci. 2019;20(13):3328. 31284572 10.3390/ijms20133328 PMC6651423

[72] Sun E, et al. The pivotal role of NF-kB in the pathogenesis and therapeutics of Alzheimer’s disease. Int J Mol Sci. 2022;23(16):8972. 36012242 10.3390/ijms23168972 PMC9408758

[73] Li J, et al. Protection of MCC950 against Alzheimer’s disease via inhibiting neuronal pyroptosis in SAMP8 mice. Exp Brain Res. 2020;238(11):2603–14. 32892233 10.1007/s00221-020-05916-6

[74] Kwak YT, Yang Y, Koo MS. Anxiety in dementia. Dement Neurocogn Disord. 2017;16(2):33–9. 30906368 10.12779/dnd.2017.16.2.33 PMC6427954

[75] Corey-Bloom J. The ABC of Alzheimer’s disease: cognitive changes and their management in Alzheimer’s disease and related dementias. Int Psychogeriatr. 2002;14(Suppl 1):51–75. 12636180 10.1017/S1041610203008664

[76] Kumar A. Editorial: neuroinflammation and cognition. Front Aging Neurosci. 2018;10:413. 30618719 10.3389/fnagi.2018.00413 PMC6297877

[77] Cheng C, et al. Targeting neuroinflammation as a preventive and therapeutic approach for perioperative neurocognitive disorders. J Neuroinflammation. 2022;19(1):297. 36503642 10.1186/s12974-022-02656-y PMC9743533

[78] Richwine AF, et al. Cognitive deficits in interleukin-10-deficient mice after peripheral injection of lipopolysaccharide. Brain Behav Immun. 2009;23(6):794–802. 19272439 10.1016/j.bbi.2009.02.020 PMC2881543

[79] Sanchez-Molina P, et al. Chronic IL-10 overproduction disrupts microglia-neuron dialogue similar to aging, resulting in impaired hippocampal neurogenesis and spatial memory. Brain Behav Immun. 2022;101:231–45. 34990747 10.1016/j.bbi.2021.12.026

[80] Chen Y, Yu Y. Tau and neuroinflammation in Alzheimer’s disease: interplay mechanisms and clinical translation. J Neuroinflammation. 2023;20(1):165. 37452321 10.1186/s12974-023-02853-3 PMC10349496

[81] Kwon HS, Koh SH. Neuroinflammation in neurodegenerative disorders: the roles of microglia and astrocytes. Transl Neurodegener. 2020;9(1):42. 33239064 10.1186/s40035-020-00221-2 PMC7689983

[82] Miao J, et al. Microglia in Alzheimer’s disease: pathogenesis, mechanisms, and therapeutic potentials. Front Aging Neurosci. 2023;15:1201982. 37396657 10.3389/fnagi.2023.1201982 PMC10309009

[83] Cai Z, Yan Y, Wang Y. Minocycline alleviates beta-amyloid protein and tau pathology via restraining neuroinflammation induced by diabetic metabolic disorder. Clin Interven Aging. 2013;8:1089–95. 10.2147/CIA.S46536 PMC3749817 23983461

[84] Yoshiyama Y, et al. Anti-inflammatory action of donepezil ameliorates Tau pathology, synaptic loss, and neurodegeneration in a tauopathy mouse model. J Alzheimers Dis. 2010;22:295–306. 20847440 10.3233/JAD-2010-100681

[85] Pascoal TA, et al. Microglial activation and tau propagate jointly across Braak stages. Nat Med. 2021;27(9):1592–9. 34446931 10.1038/s41591-021-01456-w

[86] Gamache J, et al. Factors other than hTau overexpression that contribute to tauopathy-like phenotype in rTg4510 mice. Nat Commun. 2019;10(1):2479. 31171783 10.1038/s41467-019-10428-1 PMC6554306

[87] Saha P, Sen N. Tauopathy: a common mechanism for neurodegeneration and brain aging. Mech Ageing Dev. 2019;178:72–9. 30668956 10.1016/j.mad.2019.01.007 PMC6377302

[88] Cavalier AN, et al. Protective effects of apigenin on the brain transcriptome with aging. Mech Ageing Dev. 2023;217:111889. 38007051 10.1016/j.mad.2023.111889 PMC10843586

[89] Wahl D, et al. Nontransgenic guinea pig strains exhibit hallmarks of human brain aging and Alzheimer’s disease. J Gerontol A Biol Sci Med Sci. 2022;77(9):1766–74. 35323931 10.1093/gerona/glac073 PMC9434446

[90] Wahl D, et al. The reverse transcriptase inhibitor 3TC protects against age-related cognitive dysfunction. Aging Cell. 2023;22(5): e13798. 36949552 10.1111/acel.13798 PMC10186603

[91] Leger M, et al. Object recognition test in mice. Nat Protoc. 2013;8(12):2531–7. 24263092 10.1038/nprot.2013.155

[92] Okun E, et al. Toll-like receptor 3 inhibits memory retention and constrains adult hippocampal neurogenesis. Proc Natl Acad Sci USA. 2010;107(35):15625–30. 20713712 10.1073/pnas.1005807107 PMC2932590

[93] Bernier M, et al. Age-dependent impact of two exercise training regimens on genomic and metabolic remodeling in skeletal muscle and liver of male mice. NPJ Aging. 2022;8(1):8. 35927269 10.1038/s41514-022-00089-8 PMC9237062

[94] Whitehead JC, et al. A clinical frailty index in aging mice: comparisons with frailty index data in humans. J Gerontol A Biol Sci Med Sci. 2014;69(6):621–32. 24051346 10.1093/gerona/glt136 PMC4022099

[95] Hay AJD, et al. Intranasally delivered mesenchymal stromal cells decrease glial inflammation early in prion disease. Front Neurosci. 2023;17:1158408. 37250395 10.3389/fnins.2023.1158408 PMC10213210

[96] Chen S, et al. fastp: an ultra-fast all-in-one FASTQ preprocessor. Bioinformatics. 2018;34(17):i884–90. 30423086 10.1093/bioinformatics/bty560 PMC6129281

[97] Dobin A, et al. STAR: ultrafast universal RNA-seq aligner. Bioinformatics. 2013;29(1):15–21. 23104886 10.1093/bioinformatics/bts635 PMC3530905

[98] Love MI, Huber W, Anders S. Moderated estimation of fold change and dispersion for RNA-seq data with DESeq2. Genome Biol. 2014;15(12):550. 25516281 10.1186/s13059-014-0550-8 PMC4302049

[99] Bourgon R, Gentleman R, Huber W. Independent filtering increases detection power for high-throughput experiments. Proc Natl Acad Sci USA. 2010;107(21):9546–51. 20460310 10.1073/pnas.0914005107 PMC2906865

[100] Law CW, et al. RNA-seq analysis is easy as 1–2–3 with limma, Glimma and edgeR. F1000Res. 2016;5:1408. 10.12688/f1000research.9005.1 PMC4937821 27441086

[101] Zhou Y, et al. Metascape provides a biologist-oriented resource for the analysis of systems-level datasets. Nat Commun. 2019;10(1):1523. 30944313 10.1038/s41467-019-09234-6 PMC6447622

[102] Yao Z, et al. A taxonomy of transcriptomic cell types across the isocortex and hippocampal formation. Cell. 2021;184(12):3222-3241.e26. 34004146 10.1016/j.cell.2021.04.021 PMC8195859

[103] Hu C, et al. Cell Marker 2.0: an updated database of manually curated cell markers in human/mouse and web tools based on scRNA-seq data. Nucleic Acids Res. 2023;51(D1):870–6. 36300619 10.1093/nar/gkac947 PMC9825416

