# Supplementary material for: Nanoligomers targeting NF-κB and NLRP3 reduce neuroinflammation and improve cognitive function with aging and tauopathy
Source: J Neuroinflammation. 2024 Jul 27;21:182. doi: 10.1186/s12974-024-03182-9 (PMC11283709; doi:10.1186/s12974-024-03182-9)
Supplement: Supplementary file 1 — Additional file 1: Figure S1. Nanoligomers targeting NF-κB and NLRP3 increase grip strength in old wildtype mice and have no adverse effects on overall physical health/frailty. Left: Forelimb grip strength in young, old and old Nanoligomer-treated wildtype mice, as well as littermate (LM) controls, rTg4510 tauopathy and rTg4510 Nanoligomer-treated mice. Right: Frailty Index in the same animals. N = 6–11/group; *p < 0.05. Figure S2. Nanoligomers targeting NF-κB and NLRP3 reverse age- and tauopathy-related increases in many cytokines in the brain. Multiplex ELISA analyses of prefrontal cortex tissue from young, old and old Nanoligomer-treated wildtype mice, as well as littermate (LM) controls, rTg4510 tauopathy and rTg4510 Nanoligomer-treated mice. N = 6–11/group; *p < 0.05 vs. young/LM; #p < 0.05 vs. old/rTg4510; red lines represent limit of detection. Figure S3. Nanoligomers targeting NF-κB and NLRP3 modulate glial cell morphology in old mice. Representative images from Fig. 5 (left in each panel) and corresponding images (right in each panel) showing skeletonization (white lines) for assessing morphology in A astrocytes stained for GFAP and B microglia stained for IBA1. Figure S4. Immunoblotting confirmation of hits in RNA-seq data. Raw immunoblots and quantifications showing protein levels that track with RNA-seq gene expression levels for Indoleamine 2,3‐dioxygenase 1 (IDO1) and Interleukin 17 Receptor E (IL17RE), both of which have also been linked with differences in cognitive function, in young, old and old Nanoligomer-treated wildtype mice, as well as littermate (LM) controls, rTg4510 tauopathy and rTg4510 Nanoligomer-treated mice. *p < 0.05; **p < 0.01. Note: same blot/GAPDH loading controls as in Fig. 7. [file 12974_2024_3182_MOESM1_ESM.docx]

**Supplementary Figures**

**
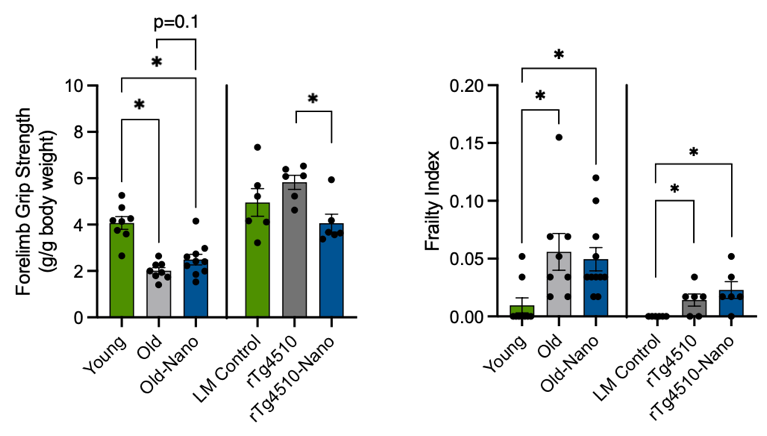
**

**Figure S1. Nanoligomers targeting NF-κB and NLRP3 increase grip strength in old wildtype mice and have no adverse effects on overall physical health/frailty.** Left: Forelimb grip strength in young, old and old Nanoligomer-treated wildtype mice, as well as littermate (LM) controls, rTg4510 tauopathy and rTg4510 Nanoligomer-treated mice. Right: Frailty Index in the same animals. N = 6-11/group; *p < 0.05.

**Figure S2. Nanoligomers targeting NF-κB and NLRP3 reverse age- and tauopathy-related increases in many cytokines in the brain.** Multiplex ELISA analyses of prefrontal cortex tissue from young, old and old Nanoligomer-treated wildtype mice, as well as littermate (LM) controls, rTg4510 tauopathy and rTg4510 Nanoligomer-treated mice. N = 6-11/group; *p < 0.05 vs. young/LM; ^#^p < 0.05 vs. old/rTg4510; red lines represent limit of detection.

**Figure S3. Nanoligomers targeting NF-κB and NLRP3 modulate glial cell morphology in old mice.** Representative images from Figure 5 (left in each panel) and corresponding images (right in each panel) showing skeletonization (white lines) for assessing morphology in **(A)** astrocytes stained for GFAP and **(B)** microglia stained for IBA1.

**Figure S4. Immunoblotting confirmation of hits in RNA-seq data.**

Raw immunoblots and quantifications showing protein levels that track with RNA-seq gene expression levels for Indoleamine 2,3‐dioxygenase 1 (IDO1) and Interleukin 17 Receptor E (IL17RE), both of which have also been linked with differences in cognitive function, in young, old and old Nanoligomer-treated wildtype mice, as well as littermate (LM) controls, rTg4510 tauopathy and rTg4510 Nanoligomer-treated mice. *p < 0.05; **p < 0.01. Note: same blot/GAPDH loading controls as in Figure 7.
